# Morphology and Species Composition of Southern Adriatic Sea Leptocephali Evaluated Using DNA Barcoding

**DOI:** 10.1371/journal.pone.0166137

**Published:** 2016-11-28

**Authors:** Alessandra Anibaldi, Claudia Benassi Franciosi, Francesco Massari, Fausto Tinti, Corrado Piccinetti, Giulia Riccioni

**Affiliations:** 1 Department of Biological, Geological and Environmental Sciences, University of Bologna, Laboratory of Marine Biology and Fishery, Fano (PU), Italy; 2 Department of Biological, Geological and Environmental Sciences, University of Bologna, Laboratory of Genetics & Genomics of Marine Resources and Environment (GenoDREAM), Ravenna, Italy; Maurice Lamontagne Institute, CANADA

## Abstract

Leptocephali are the characteristic larvae of the superorder Elopomorpha that are difficult to identify at the species level. In this study, we used DNA barcoding (i.e. short genetic sequences of DNA used as unique species tags) coupled with classical taxonomic methods to identify leptocephali in the southern Adriatic Sea. This information will provide an assessment of the biodiversity of the eel larvae in this region. A total of 2,785 leptocephali were collected, and using external morphology were assigned to seven morphotypes: *Ariosoma balearicum*, *Conger conger*, *Gnathophis mystax*, *Facciolella* sp., *Nettastoma melanurum*, *Dalophis imberbis* and *Chlopsis bicolor*. Collectively, these seven morphotypes are considered to be a good proxy for the Anguilliformes community (the main order of the Elopomorpha) in the southern Adriatic Sea (to date, seven families and sixteen species have been recorded in this region). Interestingly, the higher number of *G*. *mystax* larvae collected suggests an increased abundance of this genus. To validate the morphological identifications, we sequenced 61 leptocephali (at a 655 bp fragment from the cytochrome oxidase subunit 1 mitochondrial region) and developed barcode vouchers for the seven morphotypes. Using genetic information from reference databases, we validated three of these morphotypes. Where reference sequences were unavailable, we generated barcodes for both adult and juvenile forms to provide additional genetic information. Using this integrated approach allowed us to characterize a new species of *Facciolella* in the Adriatic Sea for the first time. Moreover, we also revealed a lack of differentiation, at the species level, between *G*. *mistax* and *G*. *bathytopos*, a western Atlantic Ocean species. Our morphological and barcode data have been published in the Barcoding of the Adriatic Leptocephali database. This work represents the first contribution to a wider project that aims to create a barcode database to support the assessment of leptocephali diversity in the Mediterranean Sea.

## Introduction

Leptocephali are the larval form shared by the superorder Elopomorpha that is a morphologically diverse group of predominantly marine teleost fishes comprising of approximately 1,000 species (eels, tarpons, bonefishes and notacanths). These species have recently been divided into four orders: [1]. Eels (order Anguilliformes), the largest of the four orders, includes at least 15 families (Anguillidae, Heretenchelyidae, Moringuidae, Colocongridae, Congridae, Derichthyidae, Muraenesocidae, Nemichthyidae, Nettastomatidae, Ophichthidae, Serrivomeridae, Synaphobranchidae, Chlopsidae, Myrocongridae, Muraenidae) and more than 800 species [2]. Several of these species have commercial and conservation interest (e.g., *Anguilla anguilla* and *Conger conger*). In the central Mediterranean Sea, nine families (20 species) have been recorded, seven of which (16 species) have been reported in the Adriatic Sea ([3]; Table A in S1 File). Anguilliform eels are found worldwide, living in a wide variety of temperate, subtropical and tropical habitats, ranging from freshwater rivers and estuaries to shallow continental shelves and oceanic depths [4,5,6]. As adults, they are characterised by an elongated, snake-like body that lacks pelvic fins. This form scarcely resembles its larval form, known as leptocephalus. In contrast, the leptocephalus is almost transparent, with a willow leaf-like body shape that is highly laterally compressed, and adapted to pelagic life. A variety of unique morphological, physiological and ecological characteristics, including a long larval phase (several months to one year), distinguish leptocephali from most other fish larvae [6]. In recent years, a number of studies considering the biology and ecology of leptocephali have been undertaken (e.g.,[6–11]); however, several aspects of their life history traits and taxonomy are still poorly understood. In particular, important achievements have been accomplished in the area of morphology and species identification thanks to surveys conducted in the Pacific (e.g., [12–15]), the Gulf of Guinea [16] and the western North Atlantic Ocean. In this latter region, many leptocephali have been identified to the species level [17,18], providing invaluable data.

While these works have made a significant contribution to the task of identifying leptocephali, their focus has been limited to the species that inhabit these specific geographical regions. In the Mediterranean Sea, the identification of some species (e.g., *Facciolella oxyrhyncha*, *Gnathophis mystax* and *Dalophis imberbis*) still depends on references such as Grassi [19] and D’Ancona [20] that date to the early 1900s. Grassi’s monograph (later reviewed by D’Ancona) provides extensive and detailed descriptions of anguilliforms in the Mediterranean Sea, describing the morphologies of ten families (Anguillidae, Chlopsidae, Congridae, Muraenesocidae, Muraenidae, Nemichthyidae, Nettastomatidae, Notacanthidae, Ophichthidae and Synaphobranchidae) during their developmental stages (from pre-larvae to juveniles). Unfortunately, this guide is out of date and furthermore, neither English language or on-line versions are available. Thus, further studies are required to obtain a more reliable and up-to-date assessment of the taxonomic composition of leptocephali in the Mediterranean Sea. A correct species identification represents one of the first steps in monitoring and conservation of marine resources and ecosystem studies [21].

Traditionally, fish identification relies heavily on the analysis of morphological characteristics and usually requires the involvement of expert taxonomists. Consequently, technical impediments can severely limit the use of this approach for early developmental stages or rare and cryptic species [22,23]. For example, fish larvae are often very small and fragile, making it difficult to distinguish the fine morphological features required for correct identification. Leptocephali are subject to continuous developmental changes, especially during metamorphosis where these changes can be drastic [6], making it incredibly difficult to identify these larvae.

To overcome these difficulties and improve overall species identification, DNA-based methods have become routinely used to support and/or integrate with classical taxonomic approaches (e.g., [24,25,26]). To date, several molecular genetic methods have been indeed successfully used to identify eels at the different life cycle stages [7, 27–30]. DNA barcoding, a method that uses a standardized, universal DNA region as a unique species tag has been particularly effective. This technique allows for rapid and accurate species identification [31,32]. It has also proven efficient in solving taxonomic ambiguities, revealing cryptic diversities [33,34] and identifying early life stages [35–38]. Despite its benefits, the use of DNA barcoding for identifying leptocephali is still in its infancy [30,39]. However, its application could address several outstanding issues, providing a tool that can: i) match leptocephali with their respective adult forms (where adults sequences are available), ii) evaluate whether similar leptocephali morphotypes represent single molecular operational taxonomic units [40] and iii) offer an alternative identification approach when specimens are severely damaged, rendering morphological identification impossible.

In this study, we aimed to improve overall identification efficiency and develop the first records of anguilliform larval assemblage in the southern Adriatic Sea by using an integrated identification approach that combined traditional taxonomic methods with the use of species specific DNA barcode vouchers.

## Materials and Methods

### Sample collection

**Fig 1:**
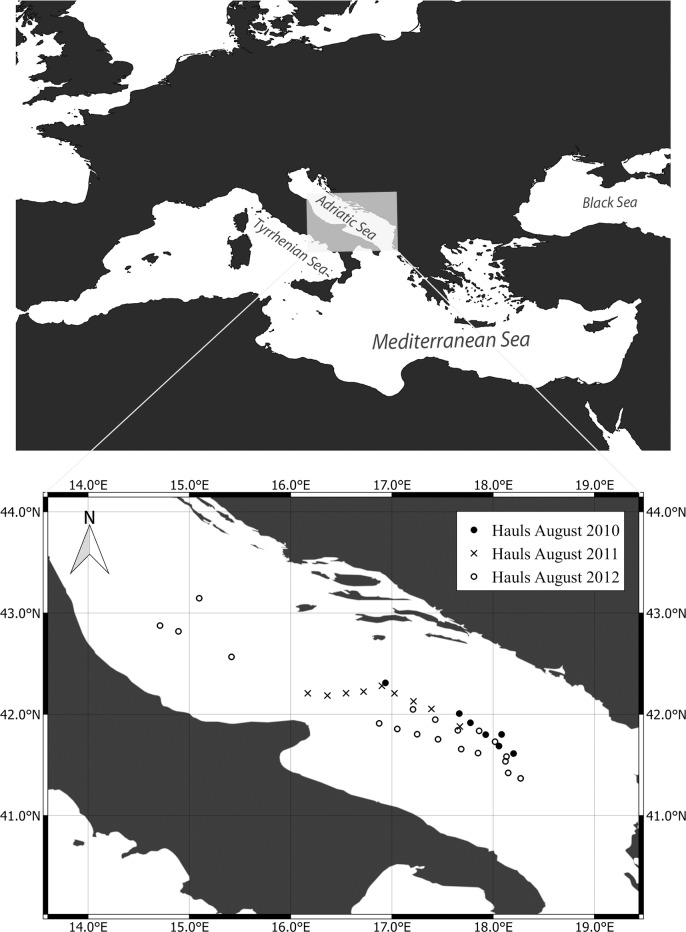
Sampling locations. Top panel: the sampling area in the Mediterranean Sea is highlighted in grey. Bottom panel: the sampling area is magnified and the sampling sites included in the three cruises are shown.

A total of 2,785 leptocephali, were collected from 35 oblique hauls during Deep Water Cruises carried out in the southern Adriatic Sea in August 2010, 2011 and 2012 by the vessel m/n Andrea (Fig 1). These hauls were performed at each station using a midwater trawl with an approximate mouth opening of 100 m^2^ and a cod-end mesh size of 20 mm. The nets were lowered to depths between 0 and 900 m, with a different maximum depth reached at each station. Specimens were fixed on board using 70% ethanol, with a subsample fixed in a 95% solution for the genetic analyses. As leptocephali are classified as zooplankton, neither special permits nor ethics approval were required for their collection.

### Morphological analyses

Total length (to the nearest 0.1 mm) was recorded for all leptocephali. To reduce the shrinkage caused by the fixation and preservation method, and to better observe the morphological features, the larvae were restored with filtered seawater prior to the measurements being taken. Larval morphology was examined using a Zeiss Stemi 2000 C stereomicroscope and compared with available dichotomous keys and descriptions for leptocephali, primarily Grassi [19] and Smith [41]. The key morphological characteristics used to identify eel larvae are body shape, the organization of the hypurals in the caudal skeleton, the length and shape of the gut (i.e., simple or with swelling or thickness), pigmentation patterns and the total number of myomeres (TMs). In specimens that were hard to identify, additional morphometric and meristic elements (e.g., head length, predorsal and preanal length, predorsal and preanal myomeres) were also considered. Specimen images were captured using a digital camera (Canon PowerShotG5) mounted on the Zeiss Stemi 2000 C stereomicroscope or using a photo scanner (Epson Perfection V600 Photo). The pictures were calibrated (with an accuracy of 0.005 mm) and handled using ImageJ software (National Institute of Health, USA, version 1.49J R). For each leptocephalus species identified, we wrote a short descriptions that focused on the key diagnostic morphological features. These descriptions were supplemented with pictures of representative individuals.

### Molecular genetic analysis

For each morphotype, one to 32 representative individuals were selected for molecular genetic analyses. This selection included also all the individuals that had been difficult to identify using the morphological characteristics. Approximately 25 mg of muscle tissue was dissected from each sample and kept in a 95% ethanol solution at -20°C until it was used. After a 30 min incubation period at 37°C to remove residual ethanol, the total genome was extracted using a NucleoSpin® Tissue Kit (MACHEREY-NAGEL GmbH & Co), following the manufacturer’s instructions. A fragment of approximately 655 bp from the 5′ region of the cytochrome oxidase subunit I (COI) gene was amplified using various combinations of the fish-specific primers described in [42]. Polymerase chain reactions (PCRs) were carried out in a total volume of 25 μL. This contained 3 μL of leptocephali DNA as a template, 0.2 μM of each primer, 1.25 U of TaKaRa Ex Taq® DNA Polymerase (Takara), 1X TaKaRa Ex Taq® Buffer and 200 μM of each deoxynucleotide solution. The thermal cycling conditions were: 1) an initial denaturation at 95°C for 2 min, then 2) 35 cycles of amplification (30 s at 94°C, 30s at 54°C and 1 min at 72°C), followed by 3) a final extension at 72°C for 10 min. Negative controls were included in each amplification reaction. All PCR reactions were performed in a TProfessional basic thermocycler (Biometra) and PCR products of the expected length size (i.e., 655 bp) were checked using 1.5% agarose gel electrophoresis after staining with GelRed™ (BIOTIUM). Products were purified with an ExoSAP-IT® kit. The products were then labeled using the BigDye® Terminator v.1.1 Cycle Sequencing Kit (Applied Biosystems, Inc.) and sequenced bidirectionally using an ABI PRISM® 3100-Avant Genetic Analyzer (Applied Biosystems). A total of 61 individual sequences, belonging to six morphotypes, were generated (Table B in S1 File). In addition, the COI sequences of a juvenile *D*. *imberbis* and an adult *F*. *oxyrhyncha* were obtained to further validate the DNA barcodes. This step was undertaken because no reference sequences were available from the public databases for these species (Table B in S1 File).

All the 63 sequences produced (61 leptocephali, one juvenile and one adult) were analysed by using, BioEdit Sequence alignment Editor [43] and MEGA 6 software [44] and were matched against the BOLD (Barcode Of Life Data system, Version 3 http://www.barcodinglife.org) and GenBank (http://www.ncbi.nlm.nih.gov/genbank/) databases to confirm their morphological identifications. We assigned each specimen to their taxonomic rank, according to their similarity values (SV): species (SV ≥ 98%), genus (92% ≥ SV < 98%) and family (≥ 85% SV < 92%). These newly generated sequences were merged and aligned with the published sequences we obtained from the databases. These results were used to build three neighbour-joining (NJ) trees [45], one for each family identified, using the uncorrected *p-*distances and 1,000 bootstrap replications generated by MEGA 6 [44]. This provided a graphical representation of the patterns of divergence between the species we analysed. A COI sequence of *Albula vulpes* (Elopomorpha: Albuliformes) was used as an outgroup in each NJ tree. Where species assignment was uncertain, we also computed the between- and within- genetic *p*-distances using MEGA 6. Finally, we performed character based species classification analyses (BLOG software; default settings; [46]) to corroborate our SV and NJ tree results.

The representative sequences and electropherograms of all the species we identified in this study, as well as information on the primers we used, were uploaded to the new barcoding database ‘Barcoding of the Adriatic Leptocephali’ (BAL) which can be accessed through the Barcode of Life Data System (BOLD, http://www.barcodinglife.org).

## Results and Discussion

Using available dichotomous keys, a total of 2,785 specimens were identified and assigned to four families and seven species of Anguilliformes (Table 1). The majority of individuals were in the larval stage (N = 2,780), with a small number in the metamorphosing stage (N = 5). The results presented below focus on individuals in the larval stage. Seven eel families are known to occur in the Adriatic Sea [3]; consequently, we felt that our study specimens were representative of the area’s eel community. Furthermore, each family was well represented, with all species belonging to the Congridae, Nettastomatidae and Chlopsidae families considered (Table A in S1 File). *Gnathophis* spp. were the most commonly identified genus, a finding that likely reflects their higher abundance in the Adriatic Sea. Note, however, that we did not have any information about their localised abundance at the sampling sites. In total, we barcoded 61 leptocephali that we also identified using morphological taxonomy. This step provided ex-novo species-specific specimen vouchers that we uploaded to the new BAL barcoding database. These sequences were, on average, 603 bp in length (Table B in S1 File). None of the amplified sequences showed insertions or deletions or stop codons, indicating that they were functional mitochondrial COI products. Meanwhile, the similarity analysis allowed us to validate, to the species level, three morphotypes (*A*. *balearicum*, *C*. *conger* and *N*. *melanurum*) with SV ≥ 98% (Table 1). For the remaining morphotypes, the limited number of DNA barcodes from voucher specimens only allowed us to confirm identity to the family level (i.e., Chlopsidae for *Chlopsis bicolor*; SV = 85.9% and Ophichthidae for *D*. *imberbis*; SV = 87%, Table 1) or the genus level (i.e., *Facciolella* sp.; SV = 93%). To address these uncertainties, we compared our leptocephali barcodes with homologous sequences obtained from an adult *F*. *oxyrhyncha* and a juvenile *D*. *imberbis* (we were unable to obtain either for *C*. *bicolor*). This enabled us to validate *D*. *imberbis* to the species-level (SV ≥ 99%; Table 1), although we could not verify the *Facciolella* sp. due to the low SVs returned against *F*. *oxyrhyncha* (SV = 93%; Table 1). With *G*. *mistax*, both the similarity search and BLOG analyses matched our specimens to the barcode voucher for *Gnathophis bathytopos* (SV = 99%; Table 1), revealing a taxonomic incongruence. As the *N*. *melanurum* sequences had not been released on the BOLD database at the point of analysis, the BLOG [46] software was only capable of confirming the identification of *A*. *balearicum* and *C*. *conger* (Table 1). Therefore, these specimens were not included in any further analysis. The NJ trees, computed using the *p-*distances instead of the Kimura-2 parameters [47], as suggested by Collins and Cruickshank [48], provided a useful description of the data (S1–S3 Figs). The following section presents a brief overview of the morphological and genetic results obtained in this study (summarized in Table 1).

**Table 1:** Summary of the morphological and molecular genetic identification of the southern Adriatic leptocephali assemblage. The morphometric and meristic counts measurements were taken from a subsample of each species: total length (TL), total number of myomeres (TM) and not assigned (NA).

Family	MORPHOLOGICAL RESULTS				MOLECULAR GENETIC RESULTS			
	Species	N	TL (mm)	TM	Similarity	%	BLOG result	NJ tree
CONGRIDAE	*Ariosoma balearicum*	39	85–152	126–130	*Ariosoma balearicum*	>98	*Ariosoma balearicum*	*Ariosoma balearicum*
	*Conger conger*	407	52–120	148–153	*Conger conger*	≥99	*Conger conger*	*Conger conger*
	*Gnathophis mystax*	2160	45–115	130–136	*Gnathophis bathytopos*	≥98	*Gnathophis bathytopos*	*Gnathophis bathytopos*
CHLOPSIDAE	*Chlopsis bicolor*	3	45–50	130–134	Chlopsidae	85	—	—
NETTASTOMATIDAE	*Facciolella* sp.	30	70–155	240–250	*Facciolella* sp.	93	NA	*Facciolella* sp.
	*Nettastoma melanurum*	7	38–98	191–210	*Nettastoma melanurum*	100	NA	*Nettastoma* sp.
OPHICHTHIDAE	*Dalophis imberbis*	139	65–197	146–160	*Dalophis imberbis* (juvenile)	≥99	NA	*Dalophis imberbis*

### *Ariosoma balearicum* (Delaroche, 1809; Congridae)

**Fig 2:**
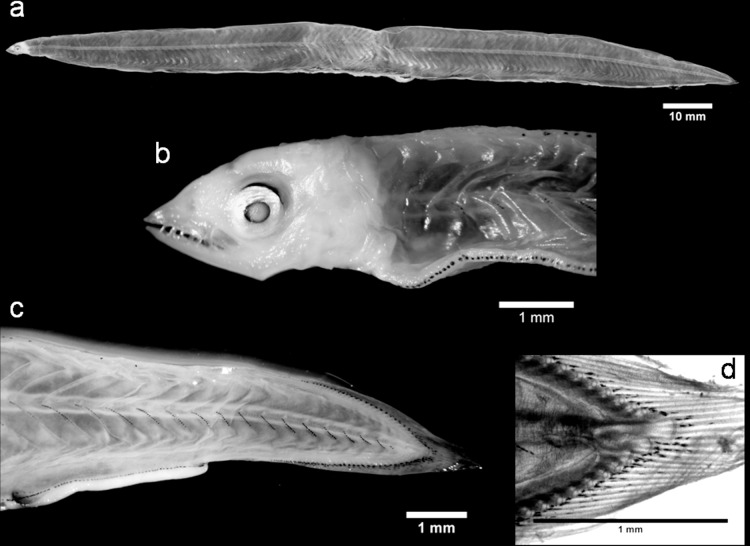
A *Ariosoma balearicum* leptocephalus: (a) lateral view of the body, (b) head region, (c) posterior region and (d) detail of the hypurals.

The leptocephali of *A*. *balearicum* (Fig 2) are one of the most collected and studied anguilliform larvae in the Atlantic and Pacific Oceans. Consequently, several morphological characterizations are available (e.g., [16,49–52]). However, *A*. *balearicum* is rarely documented in the Mediterranean Sea and the only morphological descriptions from this region are those of *Ophisoma balearicum* larvae (later *A*. *balearicum*). These were collected from the Straits of Messina and reported by Grassi [19]. Recently, Bojanić *et al*. [53] recognized some leptocephali accidentally caught in the middle of the Adriatic Sea as *A*. *balearicum*, based on their general morphology, pigmentation and morphometric characteristics. Among our specimens, *A*. *balearicum* was clearly recognizable (Fig 2; Table 1 and S1 File) as their morphology matched the descriptions (e.g., [16,19,50,51). A number of different TM ranges have been described for this species ([19]: TM = 127–136; [54]: TM = 124–136; [49]: TM = 123–131; [16]: TM = 126–138; [51]: TM = 121–136; [53]: TM = 127–133) which has resulted in a wide overall TM range (121–138). In the western North Atlantic [55], this species exhibits two TM ranges (and, accordingly, two vertebrae count ranges): a low-count form (TM = 120–130) and a high-count form (TM = 128–137). Although individuals from the Mediterranean Sea tend to belong to the latter form ([19]: TM = 127–136; [53]: TM = 127–133), the TM range identified for our specimens (TM = 126–130; Table 1) places them closer to low-count form. Nevertheless, because of this wide range, TM values are not a conclusive approach to identification. A more appropriate approach is to consider pigmentation patterns and morphological features, in combination with meristic counts and DNA barcoding. The results of all our barcode analyses clearly identified our larvae as *A*. *balearicum*. The similarity search analyses of the four COI sequences performed using the Bold System also appropriately identified these sequences as *A*. *balearicum* (SV>98%). The BLOG analysis confirmed these results. Finally, in the Congridae family NJ tree, our *A*. *balearicum* sequences were all grouped together with the published sequences (from the public database) in a unique cluster that was clearly differentiated from *Ariosoma meeki* (S1 Fig).

### *Conger conger* (Linnaeus,1758; Congridae)

**Fig 3:**
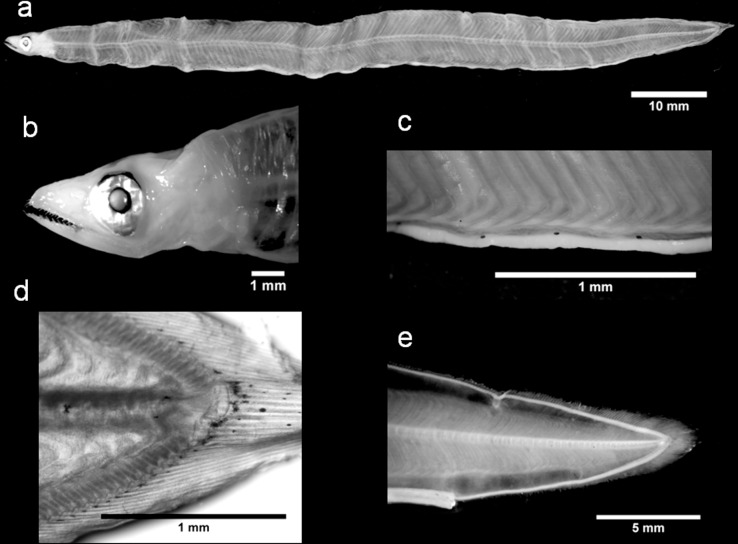
*Conger conger* leptocephali. Individual 1: (a) lateral view of the body, (b) head region, (c) abdominal pigmentation from a lateral perspective and (d) details of the hypurals. Individual 2: (e) posterior region of the body.

The *C*. *conger* (Fig 3) leptocephali we examined displayed the typical taxonomic larval characteristics, as identified by Grassi ([19], S1 File). Our TM range (TM = 148–153; Table 1) aligns with the ranges reported by Grassi ([19], TM = 148–155) and Aboussan ([56], TM = 148–153) for the larval stage of this species in the Mediterranean Sea. In contrast, the values previously identified in the Atlantic Ocean are higher (TM = 154–163; [57–61]). This discrepancy may be partially related to faulty counting techniques [58,62]. It is also possible that a distinction between high- and low-count forms, similar to that seen in *A*. *balearicum* [63], may exist. However, TM counts are not a compelling method to distinguish between different *Conger* species, i.e., the European *C*. *conger* and the American *C*. *triporiceps* share many morphological features and their TM ranges overlap. In this situation, lateral pigmentation can assume a diagnostic value since it seems to be absent in *C*. *triporiceps* leptocephali [51,59], whereas a series of stellate chromatophores are present along the lateral line of *C*. *conger* [19]. However, this characteristic requires careful interpretation as pigmentation patterns can undergo ontogenetic changes or may not be visible in poorly preserved specimens. Thus, genetic investigations have been suggested [58] and applied [29] as useful tools to support the unequivocal, systematic identification of this larvae. The results of our barcode analyses validated our morphological identification of the *C*. *conger* specimens, with all 11 barcodes achieving a SV ≥ 99%. The BLOG analysis corroborated these results and in the NJ tree, our sequences were clustered together with the public sequences in a clearly separate group, away from other *Conger* species (S1 Fig).

### *Gnathophis mystax* (Delaroche, 1809; Congridae)

**Fig 4:**
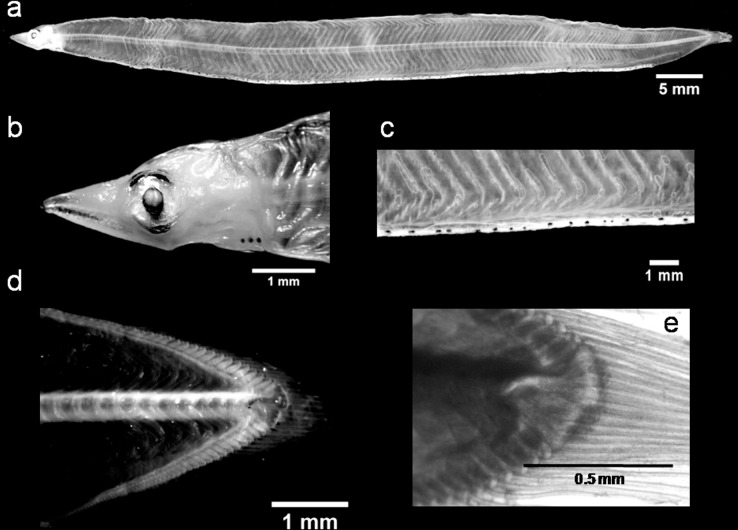
A *Gnathophis mystax* leptocephalus: (a) lateral view of the body, (b) head region, (c) abdominal pigmentation from a lateral perspective, (d) caudal region and (e) the hypurals.

The larval morphology of *G*. *mystax* (Fig 4) is similar to *C*. *conger*; however, several peculiar characteristics allow it to be easily differentiated (Table 1, S1 File). In our specimens, we found that the general morphology was comparable to Grassi's [19] description of *Congromuraena mystax* (later renamed *G*. *mystax*). One of its most distinctive morphological features is the shape of its last hypural, which shows a pronounced dorsal hump. This characteristic can be used to distinguish this species from similar *Conger* larvae. In addition, *Gnathophis* leptocephali have longer, more acute snouts [41] and a dense, double series of punctuate melanophores along the top, as compared to the *Conger* larvae. In contrast, *Gnathophis* spp. show highly similar morphologies [51,64] and their TM counts cannot be considered as a differentiating characteristic. The TM range reported by Grassi [19] for *G*. *mystax* leptocephali in the Mediterranean Sea is 133–139, but given the ranges reported by other authors (e.g., TM = 127–135 [57] and TM = 132–147 [54]), we think it should be wider (i.e., TM = 127–147). Our TM range (TM = 130–136) fell within this wider range. This widened range also overlaps with the TM ranges observed for *G*. *bathytopos* (TM = 126–141; [64]) and *G*. *capensis* (TM = 132–140; [65]) in the Atlantic Ocean. Furthermore, these species exhibit almost identical morphologies to *G*. *mystax* [51,65]. In view of these similarities, our molecular genetic analyses could provide an efficient tool to differentiate between these three species. Unfortunately, we discovered that there were no reference sequences available for any of the 32 *G*. *mystax* barcode sequences we obtained. Moreover, the blast match unexpectedly assigned these barcodes as *G*. *bathytopos* (SV ≥ 98%). The BLOG analysis results supported this preliminary result, also classifying our sequences as *G*. *bathytopos*, while the NJ tree clustered *G*. *mystax* with *G*. *bathytopos*. However, the NJ tree did differentiate this mixed group from all the other *Gnathophis* clusters (S1 Fig). The genetic distance between the two species was the same magnitude as the within species distance (*p*-distances: 0.009 vs 0.004; Table C in S1 File). While our results all suggest that our specimens were *G*. *bathytopos*, our knowledge of eel distributions suggested they were wrong. *G*. *mystax* is found in both Mediterranean Sea and the north-eastern (southern Portugal to Morocco; [66]) and western Atlantic Ocean [67] regions, whereas *G*. *bathytopos* is restricted to the western Atlantic Ocean. However, these conflicting facts lead us to draw an intriguing hypothesis: while the two species share every morphological feature, their numbers of myomeres are far from conclusive and the barcoding analyses essentially only detect one clade. Therefore, *G*. *mystax* and *G*. *bathytopos* could possibly be the same species, described in two distinct geographical regions and the morphological differences (detected mainly in the vertebrae counts between the adult forms of these two species; [68]) could merely represent a phenotypic polymorphism. Another explanation may be a lack of resolution in the barcode sequences at the species level. These hypotheses need to be considered further using nuclear sequences and further investigations on the adult forms. Overall, we found that the barcode analysis is an efficient method for differentiating *G*. *mystax* from *G*. *capensis* and *G*. *nystromi* (S1 Fig and Table C in S1 File).

### *Chlopsis bicolor* (Rafinesque, 1810; Chlopsidae)

**Fig 5:**
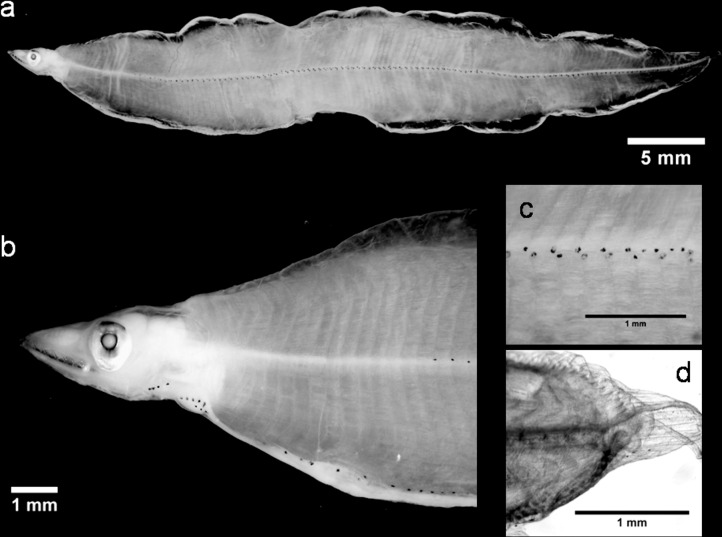
A *Chlopsis bicolor* leptocephalus: (a) lateral view of the body, (b) anterior region, (c) details of the lateral pigmentation and (d) the hypurals.

We identified three individuals as *Chlopsis bicolor*. These specimens clearly displayed the morphological characteristics of the larval form of this species, as described in the literature ([19, 69]; Fig 5; S1 File). The pre-metamorphic stage was also confirmed by the presence of pectoral fins. In *C*. *bicolor* leptocephali, these fins gradually shrink and disappear during the metamorphosis from larva to juvenile [70]. The COI sequence obtained from this leptocephalus, blasted against the BOLD database, showed the highest similarity percentage match with *C*. *bicollaris* (SV = 85%). Given this low value, we were only able to identify this specimen to the family level (Chlopsidae). As reference sequences for *C*. *bicollaris* were not publicly available and we hadn't collected any adults or juveniles to analyze, we were unable to progress this investigation any further.

### *Facciolella* sp. (Whitley, 1938; Nettastomatidae)

**Fig 6:**
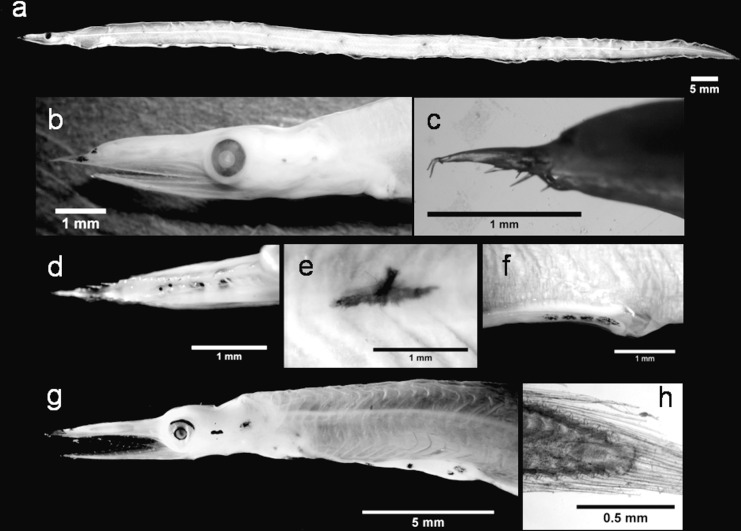
*Facciolella* sp. leptocephali. Individual 1: (a) lateral view of the body, (b) head region, (c) details of the front teeth, (d) pigmentation of the palate, (e) cross-shaped pigment along the notochord, (f) a lateral view of the pigments at the end of the gut; and Individual 2: (g) anterior region of the body, and (h) the hypurals.

Approximately 30 specimens in our collection exhibited the general morphological traits reported for the *Facciolella* larvae (Table 1; Fig 6; S1 File). For these specimens, we identified an average TM value of 244 (TM range = 240–250; Table 1). Globally, this genus is represented by six valid species, with *F*. *oxyrhyncha* the only species reported in the Mediterranean Sea [71]. It was first described from its larval stage as *Leptocephalus oxyrhynchus* by Bellotti [72] but one of the most extensive descriptions was given by Grassi [19]. This description was based on specimens that were collected in the Straits of Messina and erroneously described as *Saurenchelys cancrivora*. A further specimen has been recorded by Stramigioli *et al*. [73] in the southern Adriatic Sea. However, in spite of their similarities with *F*. *oxyrhyncha* [19], our putative *Facciolella* sp. specimens actually returned higher morphological similarity with *Saurenchelys halimyon*, another species described by van Utrecht [74]. However, there were some morpho-meristic incongruences with this species, namely its higher TM count (273). Van Utrecht [74] noted sufficient differences between his specimens and the closely related *S*. *cancrivora* (now *F*. *oxyrhyncha*), as described in Grassi [19], that they could be considered a new species of nettastomid eel. However, *S*. *halimyon* has never been formally accepted. In this regard, molecular genetic data could provide useful information. Indeed, the eight barcode sequences we analyzed only showed SVs of 92–93% with *F*. *oxyrhyncha*, *F*. *gilberti* and the COI sequence we obtained from the adult *F*. *oxyrhyncha* (EMBL accession number LT158010, http://www.ebi.ac.uk/). The BLOG analysis [46] was unable to correctly assign these specimens, while the NJ tree confirmed the similarity search results, clustering all our samples together in a well-defined molecular operational taxonomic unit. It assigned our adult *F*. *oxyrhyncha* specimen to the *F*. *oxyrhyncha* cluster (S2 Fig). The pairwise genetic distances between *Facciolella* sp. and *F*. *oxyrhyncha* and *F*. *gilberti* (0.070–0.059; Table C in S1 File) corroborated this result; they were considerably higher than the mean within-species distance of *Facciolella* sp. (0.000; Table C in S1 File). While these analyses need further validation, they do suggest the presence of a previously undescribed species in the southern Adriatic and Mediterranean Seas.

### *Nettastoma melanurum* (Rafinesque, 1810; Nettastomatidae)

**Fig 7:**
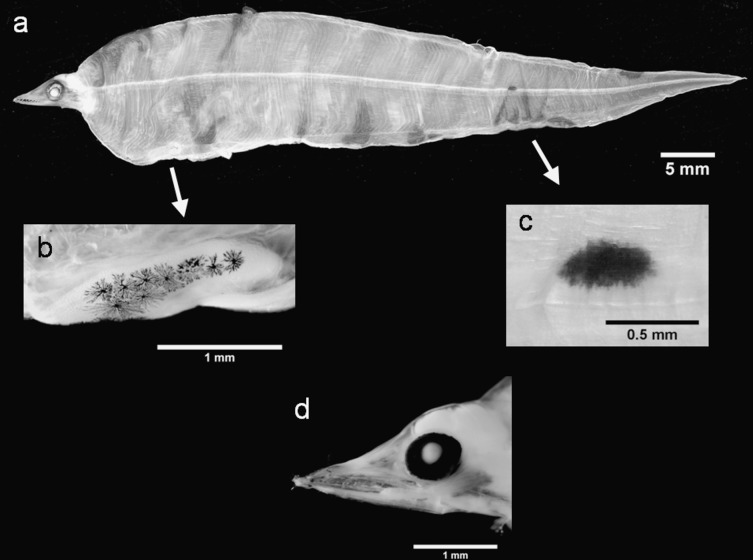
*Nettastoma melanurum* leptocephali. Individual 1: (a) lateral view of the body, (b) pigments on the gut thickening, (c) lateral pigment; and Individual 2: (d) head region.

The morphological characteristics of these leptocephali were clearly visible, enabling us to easily identify them (Fig 7; Table 1, S1 File). However, performing the meristic counts was difficult, especially in the caudal region where the myomeres were very close to each other and hard to distinguish. Nevertheless, the TM estimate (TM: ~ 200, Table 1) and the results of our broader morphological assessment corresponded to those described in the literature [16,19]. The results of the barcode analysis also confirmed this morphological assessment. Finally, our BOLD analysis matched our barcode sequence to *N*. *melanurum* voucher sequences very well (SV = 99%). However, the BLOG analysis was unable to rank our query sequence, as these voucher sequences were not publicly accessible at the time of our analysis and hence, we were unable to use them. As a consequence the NJ analysis clustered our query sequence with the congeneric species *Nettastoma parviceps* (S2 Fig).

### *Dalophis imberbis* (Delaroche, 1809; Ophichthidae)

**Fig 8:**
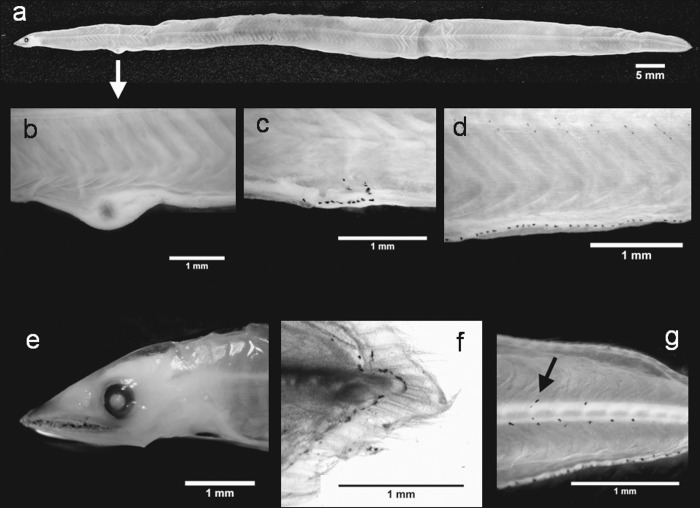
*Dalophis imberbis* leptocephali. Individual 1: (a) lateral view of the body, (b) gallbladder thickening, (c) pigments grouped on the gut, (d) pigmentation after the anus, (e) head region, (f) the hypurals; and Individual 2: (g) lateral pigmentation in the caudal region.

The armless snake eel *D*. *imberbis* belongs to the family Ophichthidae (subfamily Ophichthinae) and has been recorded in both the eastern Atlantic Ocean and Mediterranean Sea [71]. Blache [16] described several ophichthid larvae from the Gulf of Guinea, including some *Dalophis* sp. However, none of these specimens showed all the morphological characteristics of *D*. *imberbis*. Currently, the most detailed description of these leptocephali and their developmental stages at the species level can be found in [19]. Our specimens displayed complete morphological agreement with those described by Grassi [19] for *Sphagebranchus imberbis* (later *D*. *imberbis*, Fig 8; Table 1 and S1 File). Although ophichthid larvae are characterised by typical gut swellings and/or thickenings [41,56,64], this characteristic is not described for *D*. *imberbis* larvae [19]. However, gut swellings tend to disappear in the *Dalophis* larvae [16] during their development and were not visible in our specimens. The only thickening we noted in our specimens was at the gallbladder (Fig 8).

The similarity search analysis (performed on 5 barcode sequences) returned the largest SVs (87%) with representative species of Ophichthidae (*Ophichthus zophochir)* and Congridae (*Uroconger lepturus*, *Oxyconger leptognathus*). Voucher sequences for *D*. *imberbis* or other congeneric species were absent from the public databases. Accordingly, the BLOG analysis was unable to classify our query sequences. However, they almost completely matched (SV > 99%) a sequence we obtained from a juvenile *D*. *imberbis* (EMBL accession number LT158011, http://www.ebi.ac.uk/) which allowed us to confirm our initial identification. The NJ tree grouped all the *D*. *imberbis* sequences into a well-defined cluster within Ophictidae (S3 Fig). Thus, the results of the barcode sequence analyses validated our morphological identification. In addition, our *D*. *imberbis* juvenile specimen represents the first voucher sequence to be deposited in a public database.

## Conclusions

In taxonomy, identifying species using morphological characteristics is the classical approach. While they will continue to play an essential role in both identification and in developing a thorough understanding of ontogenetic changes, several issues do hamper their efficacy. The identification of anguilliform larvae, or leptocephali, is such an example where these issues can become significant. As leptocephali differ significantly from their adult forms, identifying them can be challenging, especially when considering multiple species from multiple regions. Morphological guides are available (e.g., [16,17]). However, for the larvae living in the Mediterranean, these works provide sufficient information to identify specimens to their family or genus levels, while their use in making species level identifications can be very limited. In this respect the most comprehensive descriptions are set out in Grassi [19]. To overcome morphological identification limits, molecular genetic tools, used in combination with more traditional taxonomic methods, can deliver more reliable results [75,76]. Of these genetic tools, DNA barcoding is increasingly being used to identify larval fish species [35,36,77,78], although it has rarely been used in the identification of leptocephali [30,38]. While the current lack of DNA barcodes for adult eels in public databases could limit the use of this technique for these species, nevertheless, molecular genetic data are crucial to unravel possible misidentifications (e.g., our *Gnathophis* species and [38]) or help distinguish well defined molecular operational taxonomic units (e.g., our *Facciolella* sp. specimens). It may also aide morphotype evaluation; for example, in determining whether previously identified morphotypes (using morphological tools) actually represent true species or perhaps hide cryptic species. This study demonstrates the suitability of DNA barcoding for the identification of leptocephali species across several anguilliform families and genera in the Mediterranean Sea. Future studies should seek to address the ambiguities we found in this study and continue the development of the public databases, through the contribution of more voucher specimens.

In this work, we provide the first assessment of eel diversity and abundance in the southern Adriatic Sea. We also described and photographed the morphological characteristics (validated by DNA barcoding) of *G*. *mystax*, *Facciolella* sp. and *D*. *imberbis* species. This contribution is significant as previously the only descriptions of these species appeared in the Italian monograph of Grassi [19], a reference which is only accessible to a restricted number of Italian research centres.

## Supporting Information

S1 Fig Neighbour-joining tree of the Congridae Family. Sequences obtained in this study are marked by a black diamond shape.
(TIF)

S2 Fig Neighbour-joining tree of the Nettastomatidae Family. Sequences obtained in this study are marked by a black diamond shape.
(TIF)

S3 Fig Neighbour-joining tree of the Ophichthidae Family. Sequences obtained in this study are marked by a black diamond shape.
(TIF)

S1 File Supporting information. (DOCX)
