# Supplementary figures and images for: Morphology and Species Composition of Southern Adriatic Sea Leptocephali Evaluated Using DNA Barcoding
Source: PLoS One. 2016 Nov 28;11(11):e0166137. doi: 10.1371/journal.pone.0166137 (PMC5125788; doi:10.1371/journal.pone.0166137)

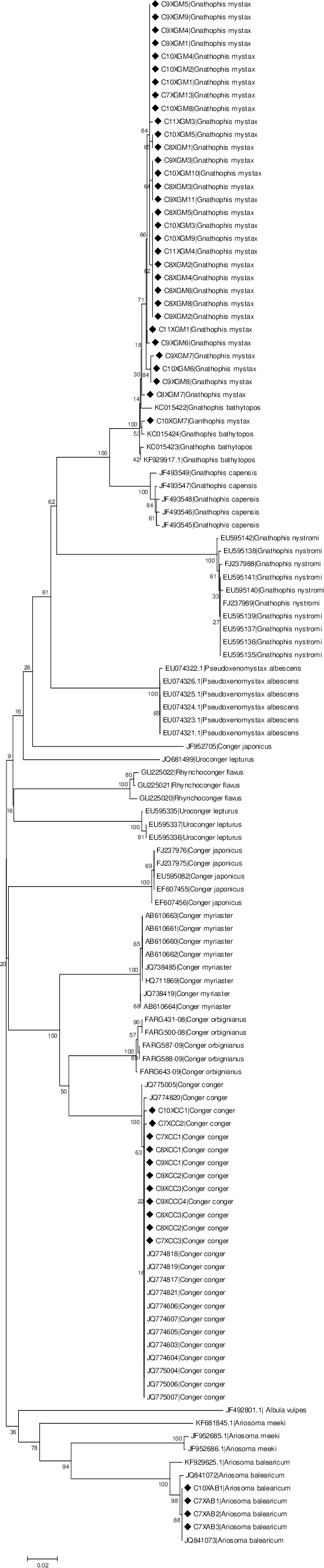

Supplement: S1 Fig — Sequences obtained in this study are marked by a black diamond shape. (TIF) [file pone.0166137.s001.tif]

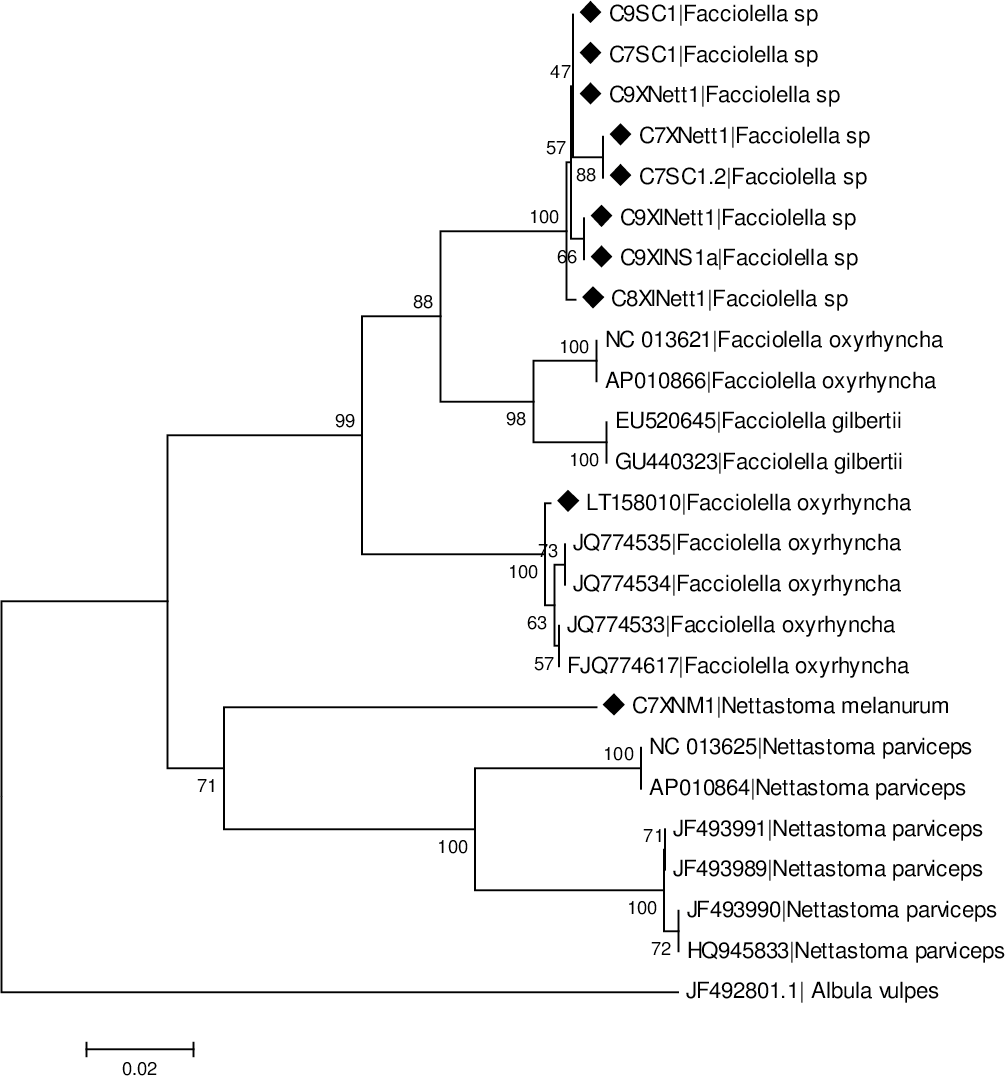

Supplement: S2 Fig — Sequences obtained in this study are marked by a black diamond shape. (TIF) [file pone.0166137.s002.tif]

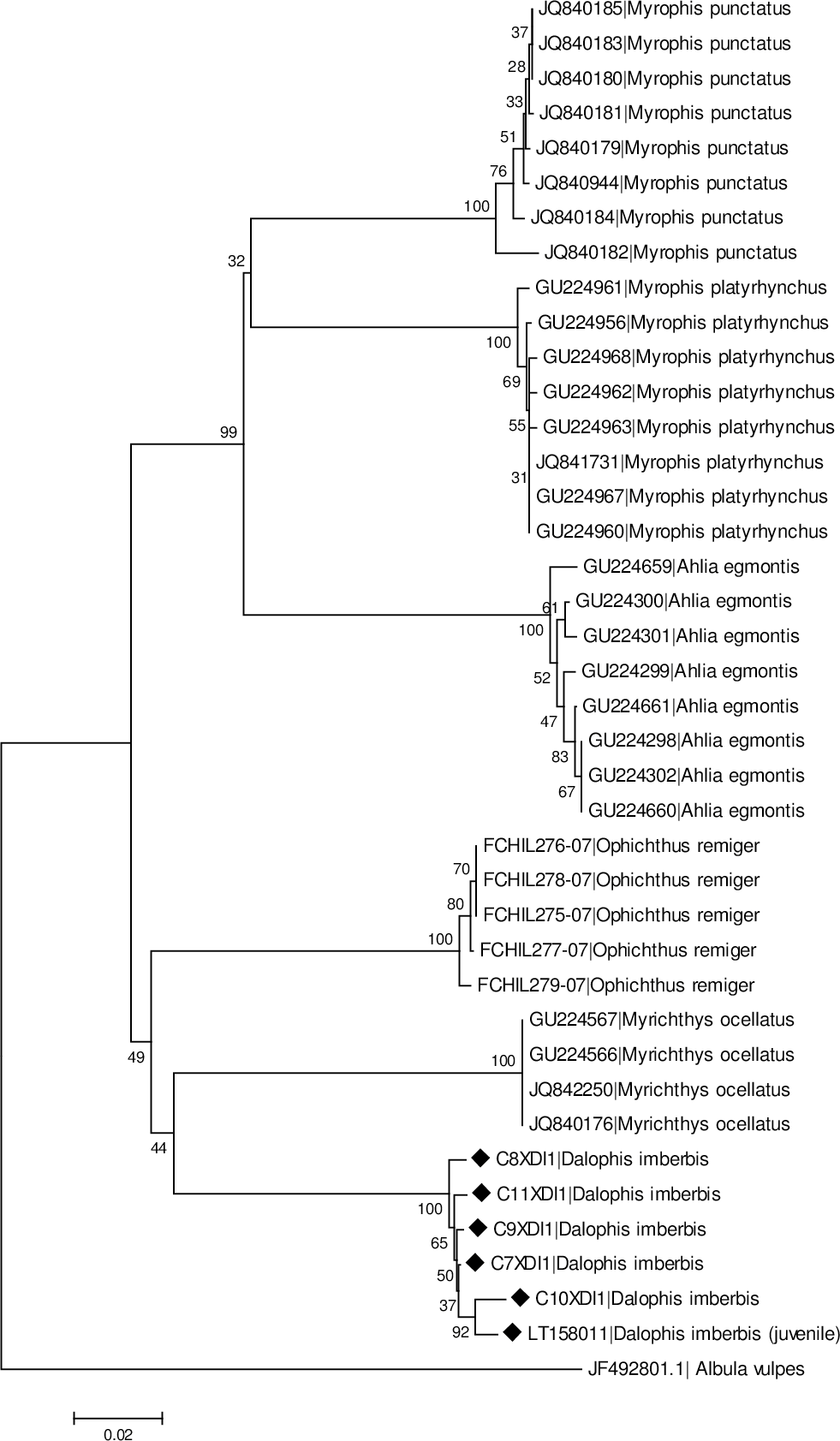

Supplement: S3 Fig — Sequences obtained in this study are marked by a black diamond shape. (TIF) [file pone.0166137.s003.tif]
