# Supplementary material for: Morphology and Species Composition of Southern Adriatic Sea Leptocephali Evaluated Using DNA Barcoding
Source: PLoS One. 2016 Nov 28;11(11):e0166137. doi: 10.1371/journal.pone.0166137 (PMC5125788; doi:10.1371/journal.pone.0166137)
Supplement: S1 File — (DOCX) [file pone.0166137.s004.docx]

**Supporting information**

Table A. **Eel families and species (order Anguilliformes) recorded in the Mediterranean Sea, the Adriatic Sea and this study.**


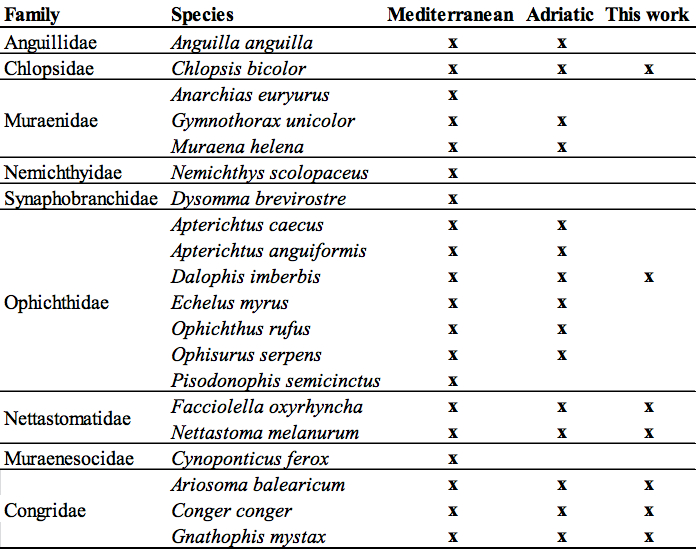


**Morphological descriptions of the leptocephali considered in this study**

***Ariosoma balearicum*** (Delaroche, 1809; Congridae)

The *A. balearicum* larvae (Fig 2) had a short head (as compared to their elongated body), subequal jaws and large and round eyes (Figs. 2a and 2b). The alimentary canal was long and straight with the anus appearing towards the very back (Figs. 2a and 2c). In the most anterior portion, a single row of very dense linear melanophores appeared along their ventral side (Fig 2b) while a double row of similar pigmentations appeared on the dorsal side of the gut, starting towards the rear and running to the anus (Fig 2c). The leptocephalus stage had short dorsal and anal fins, both originating near the posterior end of the body. Dorsal, caudal and anal fins were confluent and characterised by a row of melanophores at their base. These tended to become multiple rows in the caudal area (Fig 2c). Three and four caudal fin rays were supported by the second last and last hypural, respectively (Fig 2d). A characteristic lateral pigment, consisting of a series of small melanophores that formed short diagonal dashes on each myosepta (between the myomeres) appeared immediately below the mid-lateral line, from the head to the caudal area (Fig 2c). On the dorsal midline, a single row of dense punctuations were evident; these became increasingly sparse at the anterior and posterior ends (Figs. 2b and 2c). The total length (TL) values recorded for our specimens ranged between 85 and 152 mm and the total number of myomeres (TM) ranged between 126 and 130 (average value 127.4; Table 1).

***Conger conger*** (Linnaeus,1758; Congridae)

The *C. conger* larvae had oval eyes, elongated in a dorso-ventral direction that showed a vertical silvery band in the iris and signs of the "irido-choroid process” (Fig 3b). The gut was long and straight and almost spanned the entire length of the body (Fig 3e). Anal, dorsal and caudal fins were joined together and characterised by pigment spots at their bases (Fig 3d): massed melanophores on the caudal tip, a dense series of melanophores on the anal fin (these became a double row of sparse punctuated melanophores, towards the anterior ) and a sparse row of melanophores that were limited to the posterior region of the dorsal fin. The dorsal fin extended anteriorly and measured approximately half a body length. It originated forward of the anal fin. Pectoral fins were present and easily visible. The last hypural was quite developed, triangular and supported six rays while the second last hypural supported three rays (Fig 3d). A single row of stellate chromatophores were present along the mid-lateral line, becoming increasingly sparse as it moved towards the anterior until it disappeared (Fig 3c). The dorsal side of the alimentary canal showed a double row of punctuated melanophores, whereas the heart region was characterised by a patch of two or three melanophores. The TL values recorded for our specimens ranged between 52 and 120 mm and the TM values between 148 and 153 (Table 1).

***Gnathophis mystax*** (Delaroche, 1809; Congridae)

The *G. mystax* larvae had a sharper snout and their lower jaw showed a well-marked angle. Eyes were large and oval and displayed typical larval characteristics (Fig 4b). The gut was long and straight and almost spanned the entire length of the body (Fig 4a). It was characterised by a double series of punctuated melanophores along the dorsal side, denser than those seen in *C. conger* (Fig 4c). The dorsal and anal fins merged with the caudal fin; both were restricted to the back of the body although the dorsal fin originated in front of the anal fin. The last hypural showed a particular dorsal hump and supported 5 rays while the second last hypural had 4 rays (Figs 4d and 4e). At the base of the caudal fin rays, melanophores were scattered and visible, while they appeared in a dense row at the base of the anal and dorsal (posterior section only) fins. Pigment spots were present on the heart region (2-3; Fig 4b). The TL values recorded for our specimens ranged between 45 and 115 mm and TM values between 130 and 136 (Table 1).

***Chlopsis bicolor*** (Rafinesque, 1810; Chlopsidae)

The *C. bicolor* larvae (Fig 5) had a moderately short and deep body (Fig 5a), characterised by an elongated snout and subequal jaws. The irido-choroid process was observed (Fig 5b) and the pectoral fins were well developed. The alimentary canal was simple, without loops or thickenings and extended approximately half a body length. The dorsal fin was long and occupied about 85% of the dorsal margin. Spots of small melanophores were evident behind the lower jaw and around the heart (Fig 5b). The lateral pigmentation was confined to the midline (Fig 5a), beginning around myomere 18 (Fig 5b) and disappearing about six myomeres from the tail. This pigmentation was composed of punctuated melanophores (an average of two per myomere) arranged in an irregular double row along the notochord (Fig 5c). This became a single row towards the anterior and posterior of the body. A paired row of melanophores was visible on the dorsal side of the gut, although this was not always present at its most anterior end. An irregular paired row of minute melanophores was present on the posterior end of the anal fin, extending until the caudal fin. The hypurals were relatively enlarged with 3 rays on the second last hypural and 4 rays on the last (Fig 5d). The TL values recorded for our specimens ranged between 45 and 50 mm and TM values between 130 and 134 (Table 1).

***Facciolella* sp*.*** (Whitley, 1938; Nettastomatidae)

The *Facciolella* sp. larvae were characterised by an elongated, uniformly shallow body and a very pronounced snout (Fig 5a). The anterior extremity of the upper jaw tapered strongly, appearing as a bulge that extended beyond the lower jaw (Fig 5b). The upper jaw also supported the first pair of front teeth which were bent downward (Fig 5c). The olfactory organ extended in front of each eye for almost the entire length of the upper jaw. The nostrils were very well spaced and separated by an easily visible olfactory lamellae. In the most well preserved specimens, it was possible to identify the tubular and oval aspects of the anterior and posterior nostrils, respectively. Some pores were present along the dorsal margin of the snout, above the olfactory organ and in front of the anterior nostrils. In most specimens, the eyes appeared rounded, with silvery irises and black pigmented dorsal margins (Fig 5g). The pectoral fins were small but fairly visible and showed a dark pigmentation at their base. The dorsal fin occupied most of the dorsal region and at its most posterior part, showed well developed rays similar to those on the anal and caudal fins. The caudal fin was sharp and bore three rays on each hypural (Fig 5h). The alimentary canal extended for approximately 34% of total body length and the anus corresponded approximately to myomere 64. It was characterised by several stellate-reticulate melanophores of variable size. Some of these pigments were very large and these were evident on a thickening in the most anterior section of the alimentary canal. However, they were only evident in some individuals (Fig 5g). Smaller stellate melanophores were present on the final section of the gut, forming a double series of dots close together (Fig 5f). Along the notochord 7 or 8, deep pigment spots were visible at irregular distances and many were cross-shaped (Fig 6e). Sparse lateral spots of superficial melanophores were observed in the caudal region, just before the tail. Spots of melanophores were also present at the tip of the upper jaw (Fig 5b), deep in the olfactory organ and superficially on the palate (Fig 5d). Scattered melanophores were sometimes evident around the brain and heart regions. Finally, a characteristic linear pigment was often present behind the eye (Fig 5g). The TL values recorded for our specimens ranged between 70 and 155 mm and the TM values between 240 and 250 (average value 240; Table 1).

***Nettastoma melanurum*** (Rafinesque, 1810; Nettastomatidae)

The *N. melanurum* larvae (Fig 6) exhibited a sharp snout and a very deep body that tapered sharply at the posterior and ended with an elongated, pointed tail (Fig 6a). Their eyes were round and lacked the typical larval characteristics (Fig 6d). The pectoral fins were easily visible. The dorsal fin extended anteriorly and originated forwards of the anal fin, at short distance from the head. No dorsal, caudal or anal punctuation was evident. The alimentary canal extended almost half-way along the body and was characterised by a marked thickening that occurred approximately two-thirds of the way along its length and featured large starry melanophores (Fig 6b). Around the notochord axis, a characteristic, horizontally elongated, deep pigment spot was noted. This spot occupied approximately 3 myomeres (Fig 6c). Pigmented spots were also diffused in the head region: at the tip of the lower and upper jaws, on the bottom of the nasal cavity and adjacent to the posterior orbital rim. A deep pigmentation was evident on the esophageal area, between the eyes and the pectoral fins (Fig 6d). The TL values recorded for our specimens ranged between 38 and 98 mm and the TM average value was around 200.

***Dalophis imberbis*** (Delaroche, 1809; Ophichthidae)

The *D. imberbis* larvae showed round eyes without the typical larval characteristics, a small head and pointed snout (Fig 7). The jaws were subequal, with the lower jaw exhibiting a well-marked angle. The olfactory organ was undivided and well developed in front of the antero-superior margin of the eyes (Fig 7e). The alimentary canal extended approximately half a body length and featured a well-defined gallbladder at around myomere 23 (Figs 7a and 7b). The dorsal fin was short, originating from a very posterior position and confluent with the caudal and anal fins. The last and second last hypurals were similar, each supporting two rays (Fig 7f). A patch of very small melanophores were grouped on the gut (Fig 7c) while dense punctuation was present from the anus to the caudal end (Fig 7d). Dots and short lines of pigment were visible at the base of the caudal and anal (limited to the most posterior part) fins (Fig 7f). Some specimens also showed pigment spots at the tip of their caudal fins. The lateral pigmentation was formed by very small, punctuated melanophores arranged in short diagonal dashes on each myosepta, just below the mid-lateral line. In the caudal region, these dashes were also present above the mid-lateral line, appearing specular (Fig 7g). The TL values recorded for our specimens ranged between 65 and 197 mm and the TM values between 146 and 160.

Table B. **Number of barcoded eel specimens.**


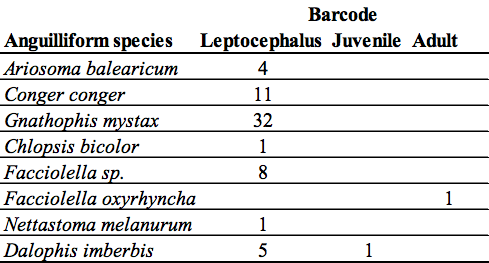


Table C. **Within- and between-species genetic *p*-distances**

|  |  |  | |
| --- | --- | --- | --- |
| **Leptocephalus species** | **Within-species** | **Between-species** | |
|  |  | *Facciolella oxyrhyncha* | *Facciolella gilberti* |
|  |  |  |  |
| *Facciolella* sp. | 0.000 | 0.070 | 0.059 |
|  |  | *Gnathophis bathytopos* | *Gnathophis capensis* |
|  |  |  |  |
| *Gnathophis mystax* | 0.004 | 0.009 | 0.058 |
